# SEOM clinical guidelines for anaplastic gliomas (2017)

**DOI:** 10.1007/s12094-017-1762-7

**Published:** 2017-10-20

**Authors:** C. Balañá, M. Alonso, A. Hernandez, P. Perez-Segura, E. Pineda, A. Ramos, A. R. Sanchez, P. Teixidor, E. Verger, M. Benavides

**Affiliations:** 1 Institut Català Oncologia Badalona, Ct. Canyet, s/n, 08916 Barcelona, Spain; 2 Complejo Hospitalario Virgen del Rocío, Seville, Spain; 3 Hospital 12 de Octubre, Madrid, Spain; 4 Hospital Universitario Clínico San Carlos, Madrid, Spain; 5 Hospital Clínic i Provincial, Barcelona, Spain; 6 Complejo Asistencial Universitario de León, León, Spain; 7 Hospital Universitari Germans Trias i Pujol Badalona, Barcelona, Spain; 8 Hospital Universitario Regional y Virgen de la Victoria, Málaga, Spain

**Keywords:** Anaplastic glioma, Anaplastic astrocytoma, Anaplastic oligodendroglioma

## Abstract

The SEOM/GEINO clinical guidelines provide recommendations for radiological, and molecular diagnosis, treatment and follow-up of adult patients with anaplastic gliomas (AG). We followed the 2016 WHO classification which specifies the major diagnostic/prognostic and predictive value of IDH1/IDH2 missense mutations and 1p/19q codeletions in AG. The diagnosis of anaplastic oligoastrocytoma is discouraged. Surgery, radiotherapy and chemotherapy with PCV or TMZ are the first-line standard of care for AG with slight modifications according to molecular variables. A multidisciplinary team is highly recommended in the management of these tumors.

## Introduction

Anaplastic gliomas constitute 15–20% of malignant gliomas [1]. They used to be included in trials together with glioblastomas, but they constitute a lower grade of malignancy with different molecular alterations and better prognosis. Median age at diagnosis is around 40 years old, 20 years lower than glioblastoma. The median overall survival (OS) is around 3.5 years being especially higher for oligodendroglial tumors where it can be as long as 13 years with proper treatment. These two factors: young adults and long overall survival force clinicians to consider long-term effects of therapy and quality of life. The identification of molecular alterations as co-deletion of chromosomes 1p and 19q and IDH1 mutations that resulted in both prognostic and predictive factors of better efficacy to treatment has substantially changed the diagnosis and the management of this disease. For these reasons, it was considered that in this updated version of the previous central nervous system tumors guideline [2], a special chapter should be dedicated to these tumors. We invite you to adopt these guidelines based on levels and grades of evidence and to spread recommendations to facilitate a correct treatment for those patients (Table 1).

**Table 1:** Summary of level of evidence and grades of recommendation

	Special situations		Level of evidence	Grade of recommendation
Surgery	Maximal tumor resection		I	A
MRI is the exploration of choice for the diagnosis and follow-up			I	A
	Baseline post-op MRI < 48–72 h		IV	A
IDH1 mutation and 1p/19q assessment are the cornestones of pathologic diagnosis			I	A
Post-operative external beam radiotherapy			I	A
Anaplastic astrocitoma	EBRT and adjuvant TMZ (12 cycles)		I	B
	Anaplastic astrocitoma IDH wild type	EBRT with concomitant and adjuvant TMZ	III	B
	Anaplastic astrocitoma NOS	RT followed by TMZ	II	B
Anaplastic oligodendroglioma 1p/19q codeleted and IDH mutated	EBRT and adjuvant PCV chemotherapy		I	A
	Elderly population	EBRT and adjuvant TMZ	II	B
Recurrent anaplastic glioma	Surgery		III	B
	Re-irradiation		III	B
	Chemotherapy TMZ/PCV/NU		II	B
	Bevacizumab		II	B

## Methodology

The Spanish Society of Medical Oncology (SEOM) and the Spanish Group for Research on Neuro-Oncology (GEINO) jointly convened an expert multidisciplinary panel in charge of a systematic review of the literature for a guidelines document on diagnoses, treatment and follow-up of anaplastic gliomas. Levels of Evidence and Grades of Recommendation were based on clinical evidence and available data in the literature. Each author approved the final version of the manuscript.

## Diagnosis

### Radiology

Imaging of the brain is ideally performed by magnetic resonance imaging (MRI) and if possible, always on the same physical MRI and with the same field strength [3] [Level I, Grade A]. The spine and cerebrospinal fluid (CSF) are not assessed in the absence of clinical symptoms.

**Fig. 1:**
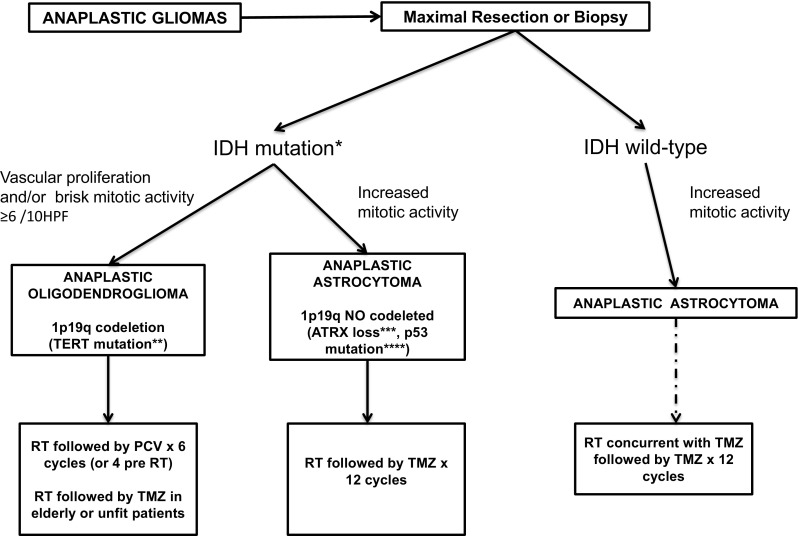
Diagnostic algorithm anaplastic gliomas *It is recommended starting with immunochemistry for R132H-mutant IDH1 followed by IDH1 and IDH2 sequencing of the tumors that were negative for IR132H-mutant DH1 immunochemistry. **Characteristic but not necessary for diagnosis. ***Recommended to confirm the astrocytic subtype but not necessary for diagnosis. Usually performed with immunochemistry. ****Characteristic but not necessary for diagnosis. *RT* radiotherapy, *PCV* procarbazine, lomustine and vincristine, *TMZ* temozolomide, *Mut* mutated

The minimum MRI adequate protocol, in terms of quality and feasibility at the majority of institutions, includes conventional MRI techniques obtained at a minimum field strength of 1.5 with the following recommended sequences: (1) precontrast 3-dimensional (3D), isotropic, IR-prepped T1-weighted (T1 W) gradient echo (IR-GRE) sequence. (2) an axial, 2-dimensional (2D) T2-weighted (T2 W) fluid-attenuated inversion recovery (FLAIR). (3) an axial, 2D, diffusion-weighted imaging (DWI); (4) an axial, 2D T2 W TSE sequence and (5) a post-contrast, 3D isotropic, T1 W IR-GRE sequence with matching acquisition parameters to precontrast T1 W images. To obtain these 3D sequences, an isotropic resolution of 1 mm × 1 mm × 1 mm is recommended with full brain coverage (Fig. 1).

Advanced MRI sequences improve accuracy over single imaging modalities. Perfusion weighted imaging (PWI) evaluates neoangiogenesis of high-grade gliomas and is able to identify areas of high-grade tumor [4]. Dynamic contrast enhancement (DCE) evaluates permeability and has shown good correlation with tumor grade and in the distinction between pseudoprogression and true tumor progression. Magnetic Resonance Spectroscopy (MRS) demonstrated moderate diagnostic performance in distinguishing high- from low-grade gliomas [5].

### Histology and molecular biology

Histology and molecular biology are necessary for a correct diagnosis of anaplastic gliomas. Three factors must be included in the pathology report: glioma subtype, grade and molecular markers according to the recent 2016 WHO classification [Level I, Grade A].

Anaplastic WHO grade III tumors are separated into two main subtypes: anaplastic astrocytomas (AA) and anaplastic oligodendrogliomas (AO). Those with uniformly rounded nuclei and perinuclear halo (“fried egg”) are considered oligodendrogliomas while those with nuclear irregularities with fibrillary processes are diagnosed as astrocytomas.

Two molecularly and virtually exclusive subtypes have been described and characterized by IDH, ATRX and TP53 mutation (astrocytic) versus IDH mutation, 1p/19q co-deletion, and TERT promoter mutation (oligodendroglial). If molecular testing cannot be performed, the term “not otherwise specified (NOS)” should be added [6]. The classification discourages the diagnosis of mixed oligoastrocytoma, since molecular studies have shown that these tumors do not constitute a separate entity [6, 7].

AA are now divided into IDH-mutant (the great majority), IDH wildtype and NOS categories. Demonstration of the ATRX mutation or loss of ATRX nuclear expression is recommended for the diagnosis of astrocytoma [8].

The diagnosis of AO requires the demonstration of both an IDH gene mutation and combined 1p/19q codeletion.

A stepwise diagnosis algorithm is recommended, starting with immunochemistry for R132H-mutant IDH1 and ATRX, followed by testing for 1p/19q codeletion, and then followed by IDH sequencing of the tumors that were negative for IDH1 immunochemistry. The combination of molecular alterations and grade III features leads to the diagnosis of AA IDH1 mutated/no mutated or AO.

## Treatment and prognosis

### Prognostic factors

The most important prognostic factors affecting outcome in patients with AG are: age (better if ≤ 50 years old), performance status (better if ≥ 70), histology (AA vs. AO), extended initial surgical resection, and molecular genetic alterations (IDH1-2, 1p/19q codeletion, and MGMT).

### Surgery

Tumor resection is one of the mainstays within the glioma WHO grade III treatment algorithm. The initial goal is to establish the appropriate diagnosis. A more extensive surgical resection is associated with longer survival, improved quality of life, reduced intracranial pressure, better tolerance to post-operative treatments and improved seizure control [9] [Level I, Grade A]. Decision of surgical resection versus biopsy depends on tumor location, size, patient age and performance status. The integration of preoperative multimodal imaging techniques into surgical procedures has increased diagnostic efficacy and reduced the risk of misclassification or under grading. The use of surgical navigation systems with functional MRI datasets, intraoperative MRI, ultrasound, intraoperative functional monitoring and the fluorescent dye 5-aminolevulinic acid to visualize tumor tissue [10, 11] helps all to increase the extent of resection, while minimizing the risk of new neurological deficits mainly in eloquent areas. The resection bed should evaluated with post-operative MRI done maximum 72 h after surgery.

### Radiotherapy

There is a Level I, Grade A recommendation for external beam radiotherapy (EBRT) after surgery in high-grade gliomas based on two randomized trials published in the 70 s, both showing increased survival with radiotherapy [12, 13]. A Canadian meta-analysis pooling six randomized trials confirmed a significant survival benefit from post-operative EBRT compared with no EBRT [14]. Many of these studies used older radiation techniques and included both grade III gliomas and glioblastoma.

The recommended dose is 59.4 Gy in 33 fractions (1.8 per fraction) or 60 Gy in 30 fractions (2.0 Gy per fraction) [15]. Treatment should be delivered with megavoltage (MV) equipment with a minimum energy of 6 MV photons. It can be done with 3D-EBRT or IMRT. CT fusion with the pre-surgical and post-operative MRI for target delineation is mandatory. Studies including brachytherapy or radiosurgery boost have not shown better outcome than EBRT alone.

For target volume and margins’ definitions, there exist differences between the Radiotherapy Oncology Group (RTOG) recommendations for USA Centers and the European Organization Research and Treatment of Cancer (EORTC) approach for European Centers. The EORTC recommends a single clinical target volume definition based on post-operative T1/T2 FLAIR abnormalities using isotropic margins without the need to cone down. This guideline takes into account the recent advantages in imaging, treatment verification and immobilization.

## First-line systemic treatment

### Anaplastic astrocytomas (AA)

#### IDH mutated

Adjuvant chemotherapy (CHT) was compared to radiotherapy in a phase III trial (NOA-04), in which patients with grade III gliomas were randomized to receive EBRT (with CHT deferred until progression), or to adjuvant CHT (with EBRT deferred until progression). CHT was based on a random assignment to either PCV (procarbazine, carmustine and vincristine) or TMZ (temozolomide) resulting in three arms after surgery: EBRT alone (arm A), PCV (arm B1), or TMZ (Arm B2). Both initial EBRT and CHT (PCV or TMZ) achieved comparable results. After a median follow-up of 9.5 years, time to treatment failure (TTF), progression-free survival (PFS), and overall survival (OS) did not show differences between arms A versus B1/B2: median TTF (4.6 vs. 4.4 years), median PFS (2.5 vs. 2.7 years), and median OS (8 vs. 6.5 years). In a subgroup analyses, patients with 1p19q-codeleted and CHT with PCV had improved PFS compared to those assigned to TMZ (9.4 vs. 4.5 years) and similar PFS compared with those assigned to EBRT (9.4 vs. 8.7 years). Median OS was 8.1 years in the group assigned to TMZ [16].

A phase III trial (RTOG 9813) compared OS for AA patients treated with EBRT with TMZ or nitrosoureas (NU). They did not found significant differences in PFS or TTF between the 2 arms. EBRT with TMZ did not appear to significantly improve OS or TTF for AA compared with RT with NU, but TMZ was better tolerated [17].

Partial results of the CATNON trial, that randomized patients to receive EBRT or EBRT and 12 cycles of TMZ or EBRT with concomitant and adjuvant TMZ or EBRT with concomitant TMZ were reported at the 2016 ASCO meeting. Newly diagnosed AA patients, who received EBRT and 12 cycles of adjuvant TMZ, had an improvement in OS [18].

Based on the results of these three trials and by the data we have so far, we recommend post-operative EBRT and TMZ treatment for 12 cycles [Level I, Grade B).

#### IDH wildtype

There are no data about the best treatment of these tumors. Some authors think they should be treated as glioblastomas because their prognosis is more similar to glioblastoma than to grade III gliomas. In the absence of data, international guides suggest to treat them with EBRT with concomitant and adjuvant TMZ or with RT and adjuvant TMZ [19] [Level III Grade B].

### Grade III gliomas NOS

This designation means that molecular diagnosis is not available because genetic testing was not performed or was inconclusive. A general consensus is to treat those patients with RT followed by CHT with either TMZ or PCV depending on the predominant morphology (astrocitic or oligodendroglial) [19] ([Level II, Grade B).

### Anaplastic oligodendroglioma (AOD)

Uncontrolled trials in the 1990s demonstrated that oligodedrogliomas were sensitive to chemotherapy, with a high response rate (70%) to PCV [20]. A long-term analysis of two large phase 3 randomized trials (RTOG 9402 and EORTC 26951) [21, 22] demonstrated a significant overall survival benefit in patients with 1p/19 codeleted anaplastic gliomas that received PCV (pre or post-radiotherapy) and EBRT versus EBRT alone. The impact on OS was not confirmed until 2013 after a median follow-up of 11 years. The RTOG 9402 randomized 291 patients (48% with 1p/19 co-deletion) and the EORTC 26951, 368 patients (25% with 1p/19 co-deletion). Both trials confirmed an increase in median OS when PCV was added to EBRT [mOS of 14.7 versus 7.3 years (HR 0.59; CI 95% 0.37–0.95; p = 0.03) and mOS not reached vs. 9.3 years (HR 0.56; CI 95% 0.31–1.03; p = 0.059)]. However, PCV CHT is a toxic chemotherapy regimen with high frequency of grade 3–4 myelotoxicity (56 and 46%, respectively) and few patients received all the planned cycles (56% in the RTOG 9402 and 30% EORTC 26951 trial). However, previous data from NOA-04 trial and retrospective series reported by Lassman et al. with more than 1013 patients [23] do not support the use of TMZ instead of PCV for the treatment of these tumors because there was a positive signal in OS and PFS favoring PCV versus TMZ in both reports. To evaluate differences on efficacy, safety profile and quality of life between PCV and TMZ on AOD, the modified CODEL trial will compare EBRT followed by PCV versus EBRT followed by TMZ.

According to these results, the standard of care for AOD after surgery is the combination of EBRT and PCV chemotherapy [Level I, Grade A].

Elderly and frail patients are under represented in both phase 3 trials. The sequential combination of RT and TMZ can be a therapeutic option in this population [Level II, Grade B].

### Treatment for recurrent disease

At progression, there are several options: second surgery, focal re-irradiation and systemic treatment with CHT. None of them has been compared with the best supportive of care.

Surgery may be considered in tumors, located in non-eloquent areas and in young patients with good Karnofsky performance status [Level III, Grade B]. Re-irradiation to small tumors has also been reported [Level III, Grade B]. Patients who have not received EBRT should receive it at recurrence with CHT according to morphologic and molecular diagnosis.

If patient relapses after EBRT and CHT, alternative CHT schemas can be used (PCV after TMZ or TMZ after PCV). If progression or recurrence is later than 6 month after the end of TMZ treatment, a rechallenge with the same drug can be indicated [24]. Other drugs as NU (carmustina, fotemustine) can also be administered [25, 26].

Patients with 1p19q codelection and IDH1 mutated tumors are always more chemosensitive [Level II, Grade B] [19].

Bevacizumab is an off-label treatment that can be eventually used as a compassionate treatment offering a 6-month PFS between 20 and 60% [Level II, Grade B] [27, 28].

### Support treatment: corticoids and anticonvulsants

Corticoids must be used at the lower dose the patient needs to control neurological symptoms of mass effect and edema. Doses must be decreased progressively as soon as possible [29]. Anticonvulsants are recommended only peri-operatively. Prophylactic treatment is not recommended in the absence of crisis [30]. If needed, enzymatic inducers are not recommended to avoid interactions with CT treatment. Fertility preservation consideration has to be applied to young patients in whom long OS is expected.

### Follow-up

The baseline imaging is the one obtained immediately after surgery. Next imaging should be performed approximately 1 month after radiotherapy. Patients should be followed then clinically and by imaging every 3 months. The same MRI protocol as described in radiology must be followed in subsequent controls. The Response Assessment in Neuro-oncology Working Group criteria (RANO) guides the follow-up for radiological and clinical assessments [31] [Level III, Grade B].

Several post-treatment imaging entities should be recognized: pseudoprogression (transient, self-resolving enhancement mediated by a CHT and radiation induced local inflammatory response) that must be differentiated from early progression; pseudoresponse: a rapid regression of enhancement, perfusion and mass effect caused by antiangiogenic drugs as bevacizumab that mimics response even if tumor persists, and late radiation injury (radionecrosis). In cases of doubtful differential diagnosis between tumor recurrence and post-treatment changes PET exam using an amino acid tracer may also be helpful [Level III, Grade B] but availability of amino acid PET is unusual [32].
